# Whole-Genome Sequencing and Analysis of Tumour-Forming Radish (*Raphanus sativus* L.) Line

**DOI:** 10.3390/ijms25116236

**Published:** 2024-06-05

**Authors:** Xenia Kuznetsova, Irina Dodueva, Alexey Afonin, Emma Gribchenko, Lavrentii Danilov, Maria Gancheva, Varvara Tvorogova, Nikita Galynin, Lyudmila Lutova

**Affiliations:** 1 Department of Genetics and Biotechnology, Faculty of Biology, Saint Petersburg State University, 199034 Saint Petersburg, Russia; wildtype@yandex.ru (I.D.); lavrentydanilov@gmail.com (L.D.); v.tvorogova@spbu.ru (V.T.); nikitagalynin@mail.ru (N.G.); la.lutova@gmail.com (L.L.); 2 All-Russia Research Institute for Agricultural Microbiology, 190608 Saint Petersburg, Russiagribemma@gmail.com (E.G.); 3 Plant Biology and Biotechnology Department, Sirius University of Science and Technology, 1 Olympic Avenue, 354340 Sochi, Russia

**Keywords:** spontaneous tumours, *Raphanus sativus*, inbred lines, genomic sequence, single nucleotide variants, *CLE*, WOX

## Abstract

Spontaneous tumour formation in higher plants can occur in the absence of pathogen invasion, depending on the plant genotype. Spontaneous tumour formation on the taproots is consistently observed in certain inbred lines of radish (*Raphanus sativus* var. *radicula* Pers.). In this paper, using Oxford Nanopore and Illumina technologies, we have sequenced the genomes of two closely related radish inbred lines that differ in their ability to spontaneously form tumours. We identified a large number of single nucleotide variants (amino acid substitutions, insertions or deletions, SNVs) that are likely to be associated with the spontaneous tumour formation. Among the genes involved in the trait, we have identified those that regulate the cell cycle, meristem activity, gene expression, and metabolism and signalling of phytohormones. After identifying the SNVs, we performed Sanger sequencing of amplicons corresponding to SNV-containing regions to validate our results. We then checked for the presence of SNVs in other tumour lines of the radish genetic collection and found the *ERF118* gene, which had the SNVs in the majority of tumour lines. Furthermore, we performed the identification of the *CLAVATA3/ESR* (*CLE*) and *WUSCHEL* (*WOX*) genes and, as a result, identified two unique radish *CLE* genes which probably encode proteins with multiple CLE domains. The results obtained provide a basis for investigating the mechanisms of plant tumour formation and also for future genetic and genomic studies of radish.

## 1. Introduction

Tumour formation is a pathological process that results from the uncontrolled proliferation of a group of cells. Tumours occur in virtually all multicellular organisms and are represented by newly formed tissues whose cells are actively proliferating. In animals, a system of proto-oncogenes and tumour suppressor genes forms a complex network that systemically controls the rate of cell division, growth, and differentiation at the level of the whole organism. Disruption of this control, both under the influence of environmental factors and due to genomic instability, leads to the development of tumours. Higher plants contain functional orthologues of many mammalian tumour suppressors and oncogenes, but mutations in these genes in plants have not led to tumour formation, suggesting a very different principle of organisation of the systemic control of cell division and differentiation in plants [1,2,3]. 

Most examples of plant tumours arise as a result of interactions with a variety of pathogens and phytophages, from bacteria and viruses to nematodes and arthropods [4]. The development of pathogen-induced tumours is usually associated with an imbalance of such phytohormones such as auxin, cytokinin, and peptide hormones [4]. More rarely, spontaneous tumours develop in the absence of pathogens in the plants with specific genotypes (mutants, interspecific hybrids, inbred lines), making them more similar to animal tumours [1,4]. The exact causes of spontaneous tumour formation have only been studied in *Arabidopsis* mutants that have defects in cell–cell adhesion due to the loss of function of enzymes involved in the biosynthesis or modification of cell wall components [5,6,7,8]. The study of these mutants has revealed one of the unknown aspects of the systemic control of cell division in plants, bringing cell adhesion to the fore [1]. At the same time, not all tumour mutants of *Arabidopsis* and other plants have impaired cell adhesion. Studying other examples of plant tumours may help in identifying other systemic regulators of cell division in higher plants. 

**Figure 1:**
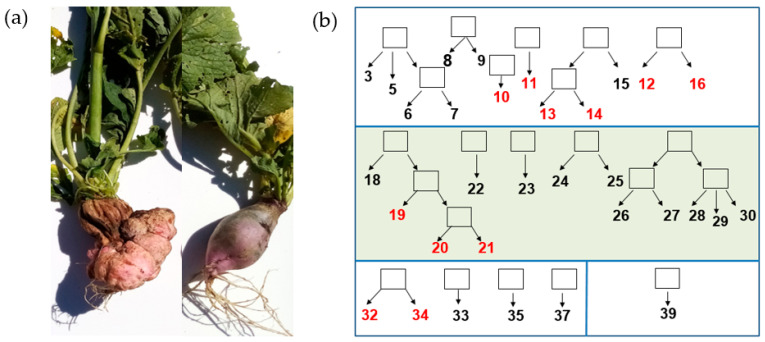
Spontaneous tumour formation in inbred radish lines: (**a**). taproots of related lines 19 (left) and 18 (right) contrasting in the tumour formation trait; (**b**). a family tree of the radish genetic collection showing the origin of the inbred lines; tumour lines 10, 11, 12, 13, 14, 16, 19, 20, 21, 32, 34 are marked in red; the squares indicate the intended progeny of each radish line. Different boxes represent lines of diverse cultivars. The sector that includes lines originating from the Saxa cultivar is highlighted in green.

The objects of our research are spontaneous tumours of the inbred lines of European cherry radish (*Raphanus sativus* var. *radicula* Pers.) (Figure 1a). The genetic collection of radish inbred lines has been maintained at St. Petersburg State University (SPbSU) since the 1960s by selfing individual plants, and now contains thirty-three highly inbred lines, originated from four radish cultivars. Eleven radish inbred lines stably form tumours on the taproots of plants in the flowering stage ([9,10], Figure 1). 

As with most examples of spontaneous tumours in plants, the mechanism triggering tumour formation in the radish inbred lines is unknown. Tumours on radish taproots originate from the pericycle and cambium as callus-like structures and later acquire features of secondary differentiation, such as vasculature, and meristematic foci similar to root apical meristems due to auxin maxima and *WOX5* expression [11]. The RNA-seq of radish tumours compared to lateral roots revealed the differential expression of the more than 1600 genes [12]. Most of the pathways upregulated in radish tumours were associated with the control of cell division, showing the extreme activation of this process in the tumour tissue [12].

In the present work, we have sequenced the genomes of two closely related radish inbred lines 18 and 19 that contrastingly differ in their ability to form tumours ([9], Figure 1). In genetic crosses between these two lines, this trait was inherited as a monogenic recessive [13], providing an opportunity to identify a specific gene that regulates spontaneous tumour formation. 

As a result, a number of SNVs (InDels and SNPs) were revealed in the tumour radish line. Among these, we found more than a hundred SNVs in the CDS of protein-coding genes that are thought to lead to changes in protein structure (“stop lost”/“stop gained” or a frameshift) or in positions 1–20 of the 5’-UTR, which could severely affect the translation efficiency [14]. Many of the genes with such SNVs in the tumour line are homologues of *Arabidopsis* genes, which are involved in cell cycle regulation, cytoskeleton organisation, meristem development, and phytohormone homeostasis. Among these, we selected 108 SNVs that are in the homozygous state in the tumour radish line. The presence of the selected InDels and SNPs in the radish tumour line was verified by sequencing the amplicons of the corresponding gene regions in radish lines 18 and 19. 

To search for the association of SNVs with spontaneous tumour formation, we performed the sequencing of 40 SNV-containing gene regions in seven tumour and fourteen non-tumour radish lines of the SPbSU genetic collection. As a result, we found that the *RsERF018* gene contains the CAG insertion in the 5’-UTR close to the start codon in most tumour radish lines and only two non-tumour lines, which allows us to propose it as a candidate regulator of spontaneous tumour formation. 

Based on genome assemblies’ data of two radish inbred lines, we carried out the identification and chromosomal localisation of the genes belonging to the *CLE* and *WOX* families which are known to be master regulators of meristem identity and stem cell homeostasis. Among them, we identified new, previously uncharacterised radish *CLE* genes which are likely to encode proteins with multiple CLE domains. Homologues of such a group of *CLEs* are absent in *Arabidopsis*, but have been identified in *Brassica napus* [15].

The sequencing of the genome of the tumour radish line may be a step towards identifying new mechanisms underlying spontaneous tumour formation in higher plants.

## 2. Results

### 2.1. Assessment of the Assembly Quality of the Genomes of Two Radish Inbred Lines

To compare the genomic DNA sequences of tumour radish line 19 and non-tumour radish line 18, we performed a hybrid chromosome-level assembly using a combination of data obtained by Illumina and Oxford Nanopore sequencing methods.

**Figure 2:**
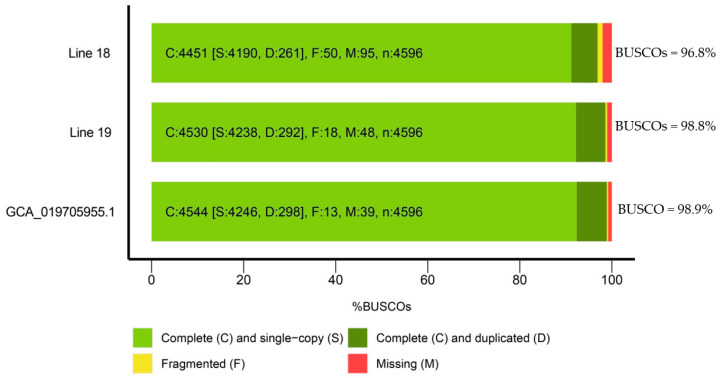
Comparative characteristics of the genomes of radish lines 18 and 19 sequenced in this work and the radish reference genome (GCA_019705955.1). The analysis was carried out using the BUSCO programme.

As a result of the assembly quality assessment using the BUSCO programme (https://busco.ezlab.org/ (accessed on 30 May 2024)), it was shown that the number of single copies of nuclear genes was greater than 92.2% for line 19 and 91.1% for line 18. The number of duplicated sequences was 6.4% for line 19 and 5.7% for line 18, and the overall assembly quality index was greater than 98.6% for line 19 and 96.8% for line 18, indicating a low content of fragmented or incomplete sequences and no contamination by sequences from other phylogenetic taxa (Figure 2). The assembly parameter values obtained using the Quast programme indicated that the genome size of line 18 was 492,907,896 bp with N50 = 12,750, and the genome size of line 19 was 480,234,765 bp with N50 = 13,846,043. These parameters are comparable to the characteristics of reference radish genomes [16,17,18].

A BUSCO analysis of the genome assemblies of lines 18 and 19 showed quality indicators of 96.8 and 98.8, respectively. Thus, the assembly quality of the genomes of radish lines obtained in this work is not inferior to those available in the NCBI database (https://www.ncbi.nlm.nih.gov/datasets/genome/?taxon=3725, accessed on 23 January 2024). 

### 2.2. Identification of SNVs in the Protein-Coding Genes of the Tumour Radish Line

By analysing the genome sequences of tumour and non-tumour radish lines, we have identified a large number of SNVs (514,083 InDels and 2,260,270 SNPs) in tumour line 19 (Table 1, Supplementary Figure S1). Among them, 35,399 InDels and 688,148 SNPs were located in the CDS of protein-coding genes or in positions −1–20 of the 5’-UTR. Of these, 21,698 InDels and 9451 SNPs were likely to result in the altered translation of the corresponding proteins due to a frameshift, loss of a start or stop codon, gain of a start codon, or decrease in the translation efficiency due to the changes in the 5’-UTR near the start [14].

Among the genes with these SNVs, we selected 240 InDels and 135 SNPs in the genes related to GO that are probably associated with the control of plant cell proliferation: related to the regulation of cell growth (GO:0008283, GO:0007346, GO:0010564, GO:0000278, GO:0051726, GO:0006261, GO:0042023, GO:0000910, GO:0000911, GO:0000226, GO:0009828, GO:0009505, GO:0009825), meristem activity (GO:0010014, GO:0010075, GO:0009933), phytohormone signalling (GO:0009736, GO:0009690, GO:0009686, GO:0045487, GO:0009734, GO:0009733, GO:0009735, GO:00097390, gene expression regulation (GO:0003700, GO:0006306, GO:0034968, GO:0051567), and organogenesis (GO:0048364, GO:0048527, GO:0090451). 

**Figure 3:**
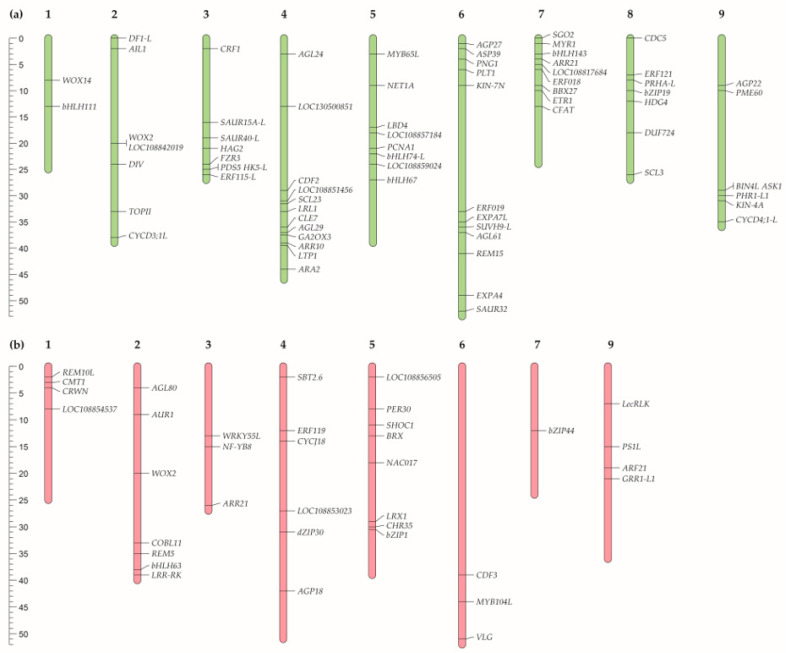
Chromosomal location of radish genes with (**a**) InDels or (**b**) SNPs identified in tumour line 19 compared to non-tumour line 18 performed using the MapChart 2.32 software (https://www.wur.nl/en/show/mapchart.htm (accessed on 30 April 2024)).

Among the genes belonging to these GO pathways, 72 genes with InDels and 36 genes with SNPs were in the homozygous state in radish line 19. Of these 72 InDels, 57 resulted in a frameshift, 9 in a frameshift and loss of a start codon, 5 in a frameshift and gain of a stop codon, and 1 in a change in the 5’-UTR near the start codon. Of the 36 SNPs, 23 resulted in a gain of a stop codon, 10 in a loss of a stop codon, and 3 in a loss of a start codon. We determined the chromosomal location of genes with such SNVs (Figure 3). More detailed information on these genes can be found in Supplementary Tables S1 and S2. 

It can be assumed that the abovementioned SNVs could lead to a loss of function of the corresponding protein-coding genes in the tumour radish line, and thus each of these SNVs could cause tumour formation. The effects of loss-of-function mutations in some of these genes on plant development have also been described for their homologues in *Arabidopsis* (Supplementary Tables S1 and S2).

### 2.3. Search for the Presence of Identified SNVs in the Tumour and Non-Tumour Lines of the Radish Genetic Collection

To search for probable candidate regulators of spontaneous tumour formation among the genes containing selected SNVs in line 19 and to verify the Nanopore and Illumina sequencing data, we amplified the corresponding gene regions of several other tumour (12, 13, 14, 16, 20, 21, 32) and non-tumour (3, 5, 6, 8, 9, 23, 25, 26, 27, 28, 29, 30, 37, 39) lines of the radish genetic collection. 

**Figure 4:**
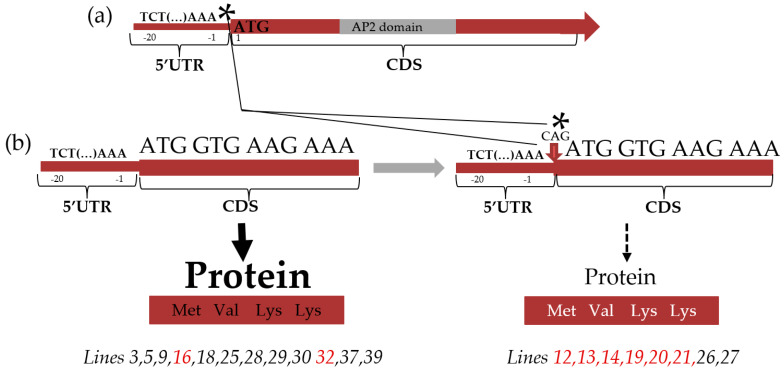
Schematic representation of the insertion (marked with an asterisk) detected in the *RsERF018* gene. (**a**) The scheme of an *ERF18* gene. The insertion is located on the border of the 5’-UTR and the start codon. (**b**) 5’-UTR insertion of the *RsERF018* gene in radish lines and its possible consequences. The amino acid content of the protein synthesised during translation of the normal sequence is marked in black, and the protein synthesised during translation in the case of the CAG insertion is marked in white. Radish tumour lines are highlighted in red.

As a result, the presence of the same SNV was confirmed in most tumour lines for the *RsERF018* gene (Figure 4). For the other 39 genes, in some of them, SNVs were only identified in line 19, or there was a polymorphism that was not associated with the tumour formation trait. 

The *RsERF018* gene, whose homologue in *Arabidopsis* controls ethylene response and cambium cell division [19], contains a CAG insertion just upstream of the start codon of the gene in tumour lines 12, 13, 14, 19, 20, and 21, and also in non-tumour lines 26 and 27, whereas no insertion was detected in tumour lines 16 and 32, as well as in most non-tumour lines (Figure 4). According to data obtained in *Arabidopsis*, this type of change in positions -1–20 of the 5’-UTR dramatically decreases the efficiency of translation [20].

The *RsERF018* gene needs to be further investigated as a possible regulator of spontaneous tumour formation.

### 2.4. Identification and Chromosomal Localisation of WOX and CLE Genes in the Obtained Genome Assemblies of Inbred Radish Lines

Meristem regulators are known to be involved in the control of the plant cell division plan, and have been shown to be involved in the development of numerous examples of plant tumours [4]. The balance between cell division and differentiation in various plant meristems is controlled by the WOX-CLAVATA system, a highly conserved regulatory module [21], consisting of CLAVATA3/EMBRYO SURROUNDING REGION-related (CLE) peptides; the protein kinase receptors that bind CLEs; and the targets of CLE action, the WUSCHEL-RELATED HOMEOBOX (WOX) homeodomain transcription factors [22,23,24].

**Figure 5:**
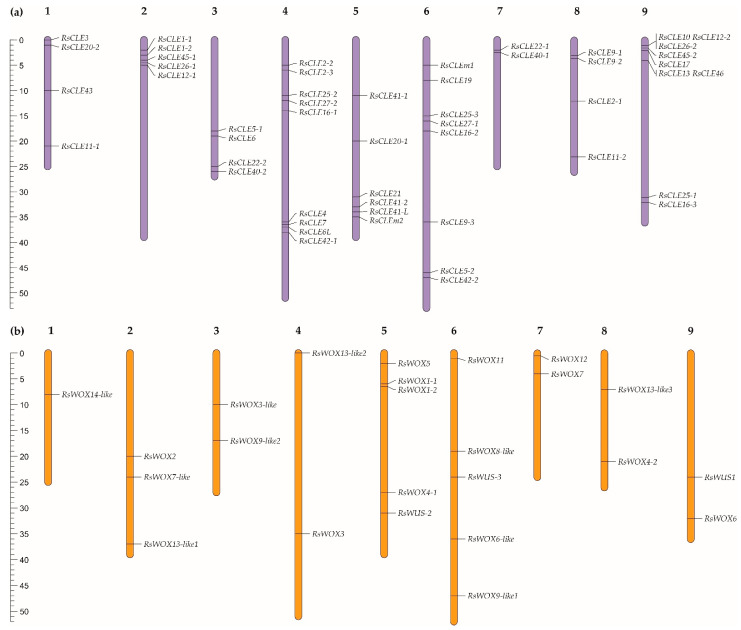
Chromosomal location of radish (**a**) *CLE* and (**b**) *WOX* family genes performed using the MapChart 2.32 software (https://www.wur.nl/en/show/mapchart.htm (accessed on 30 April 2024)).

We carried out the identification of the radish *CLE* and *WOX* family genes in our genome assemblies of 18 and 19 radish lines (Figure 5, Supplementary Figures S2 and S3). Totals of 52 *RsCLE* genes and 24 *RsWOX* genes were found. All 24 *RsWOX* genes have been identified previously [25]. Among the *RsCLE* genes, 16 *RsCLEs* were identified in our previous work [26], and other *RsCLE* genes were annotated in the reference radish genome [16]. The chromosomal location of *RsWOX* and *RsCLE* genes (Figure 5) revealed the clusters of closely located *RsCLEs* on 2, 4, and 9 radish chromosomes.

It is important to note that the *RsWOX2*, *RsWOX14,* and *RsCLE7* genes were among those identified in radish tumour line 19 as possessing SNVs in the CDS that are likely to result in loss of function (Supplementary Figure S4, Tables S1 and S2). At the same time, these SNVs were only confirmed in tumour line 19 and not in other radish tumour lines. 

### 2.5. Identification of Radish CLE Genes Likely to Encode Proteins with Multiple CLE Domains

**Figure 6:**
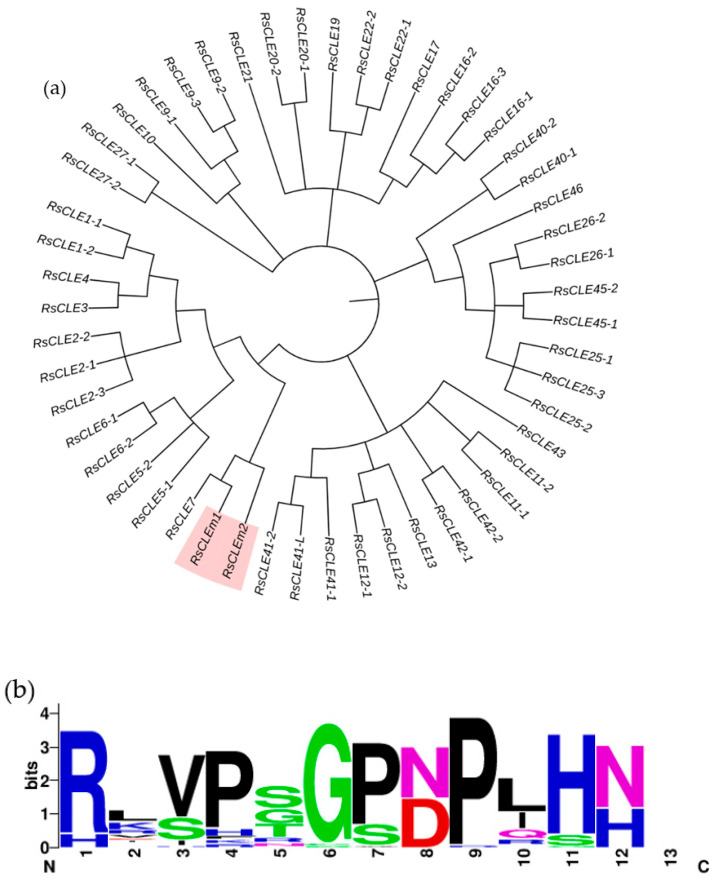
Radish *CLE* gene family (*RsCLEs*). (**a**) Phylogenetic tree of *RsCLE* genes constructed using the Neighbour-joining algorithm. The colour indicates *RsCLEm1* and *RsCLEm2* genes, which encode proteins with multiple CLE domains. (**b**) CLE domain consensus sequences of all RsCLE peptides identified in radish.

Among all the *RsCLE* genes identified in this work (Figure 6), we have found two unique *RsCLEs* of unknown function that probably encode proteins with multiple CLE domains. We then found the same genes in the radish reference genome, where they had not been described as *CLE* genes and were named in the NCBI database as actin-binding protein wsp1-like (LOC108807713) and proline-rich receptor-like protein kinase PERK10 (LOC108858878). We have uploaded the sequences of these genes found in our assemblies to the NCBI database (Submission ID: 2791313, GenBank numbers PP236904.1 and PP236905.1) under the names *RsCLEm1* and *RsCLEm2* (“*RsCLE multidomain*”). 

**Figure 7:**
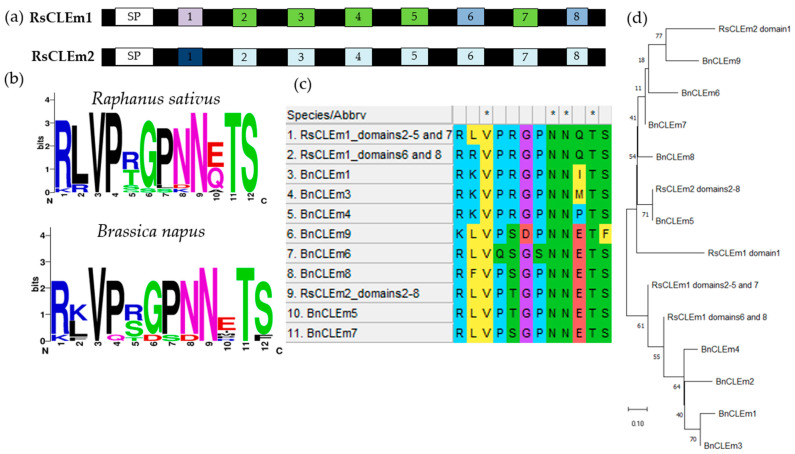
The proteins with multiple CLE domains probably encoded by *RsCLEm* genes. (**a**) A representation of the domain organisation of RsCLEm proteins, including the positions of signal peptide (SP) and CLE domains. Signal motifs were predicted with the SignalP-6.0 tool (https://services.healthtech.dtu.dk/service.php?SignalP (accessed on 30 April 2024)). Identical sequences of CLE domains are marked with the same colour. (**b**) CLE domain consensus sequences of *Raphanus sativus* and *Brassica napus*. (**c**) Sequence alignment of the putative 12-amino-acid CLE domain sequences encoded by the *CLEm* genes of *Raphanus sativus* and *Brassica napus*. (**d**) Phylogenetic analysis of the BnCLEm and RsCLEm peptides.

Each of the *RsCLEm* genes contains eight tandem CLE domain sequences separated by short spacers (Figure 7). The *CLE* genes encoding multidomain CLE proteins were previously identified and functionally studied in *Brassica napus* [15], but were absent in *Arabidopsis*.

## 3. Discussion

To date, radish genome sequencing has previously been performed for several Asian and European cultivars and isolates [16,17,27,28,29,30,31]. Polymorphism in radish is being actively studied [32,33]. The Rs1.0 genome, which is a reference genome for radish, was based on the chromosome sequences of *R. sativus* of the Korean cultivar WK10039 [16]. 

In our work, we have sequenced the genomes of two closely related radish inbred lines that differ in their ability to spontaneously form tumours [9,10,11,12,13]. This is the first attempt to sequence the genome of plants with spontaneous tumour formation.

To date, the most well-studied examples of spontaneous tumours in higher plants are several monogenic mutants of *Arabidopsis* [5,34,35,36] and one of *Nicotiana tabacum* [37], which form tumours on different organs of seedlings. In most cases, tumours in these mutants are the result of a loss of function of pectin metabolism genes, which are involved in cell wall formation and cell adhesion [5,6,7,8]. The discovery of such mutants showed that cell adhesion is one of the mechanisms that systemically regulate cell proliferation in the plant body. However, cell adhesion is not the only mechanism of such systemic regulation. In *Arabidopsis*, there are also tumour-forming mutants with loss of function of the other genes whose association with tumour development is much less obvious, such as the gene-encoding protein of the immunophilin family [34], the tyrosine phosphatase-like protein [38], and the chromatin remodelling factor [39]. Thus, the identification of plant genes whose loss of function leads to spontaneous tumour formation will help in identifying new systemic mechanisms for cell division control in higher plants.

In our work, we have identified numerous SNVs, including those in the CDS or positions −1–20 of the 5’-UTR of protein-coding genes, that distinguish the tumour radish line from the relative non-tumour line. Therefore, we can assume that certain SNVs may be inducers of spontaneous tumour formation. According to data on transcriptome analysis of the roots and spontaneous tumours in the radish inbred line, all 108 genes with loss-of-function SNVs in tumour line 19 were expressed in radish taproots [12]. Moreover, five genes with such SNVs identified in this study were among the DEGs: the expression levels of the cell cycle regulator *RsPCNA1* and the gene of unknown function *LOC108817684* were increased in the tumours, whereas the expression levels of the radish homologues of the auxin response gene *RsSAUR32*, the ethylene response cambium-associated genes *RsERF018* and *RsERF019*, and also the *RsLRR-RK* gene encoding receptor-like protein kinase were decreased [12].

Due to the large number of SNVs identified, it is currently not possible to make clear assumptions about the role of each SNV in spontaneous tumour formation. Additional testing for the presence of the identified SNVs in tumour and non-tumour radish lines revealed that a CAG insertion at position −1 of the 5’-UTR of the *RsERF018* gene was present in the seven out of eight tumour radish lines tested and was absent in the thirteen out of fifteen non-tumour lines. Without the insertion, this region contained an AAA sequence just before the start codon, which should result in high translation efficiency [20]. Therefore, an insertion of a CAG between the start and the AAA region (Figure 4) should result in a considerable decrease in the amount of the translated protein, as has been shown in *Arabidopsis* [20].

In this work, we also characterised and chromosomally localised genes of the *WOX* and *CLE* families in the genomic sequences of radish lines from the SPbSU genetic collection. Among the *RsWOX* and *RsCLE* genes, the loss-of-function SNVs were detected in the *RsWOX14*, *RsWOX2,* and *RsCLE7* genes in line 19 (Supplementary Figure S4). 

In *Arabidopsis*, the *WOX14* gene is a regulator of cambium and xylem balance and acts redundantly with the *WOX4* [40]. The *WOX2* is known to be a regulator of early embryogenesis and callus formation [41]. The *CLE7* gene in *Arabidopsis* also functions as a regulator of callus formation and regeneration [42]. Since, according to our previous data, spontaneous tumours on radish taproots originate from the cambium and develop as undifferentiated callus-like structures [11], these genes are perspective candidates for tumour regulators. However, the results on these were not very encouraging, as our data show that none of the corresponding SNVs were found in the sequences of these genes in the other radish tumour lines studied. 

The genes *RsWOX14*, *RsWOX2,* and *RsCLE7* are represented by a single copy in the radish genome, but homozygosity for the loss-of-function mutations in them does not result in reduced viability of radish line 19. According to available data, a single mutation in each of these genes in *Arabidopsis* does not cause any serious developmental abnormalities in the mature plants [40].

Analysis of the genomes of the radish lines also allowed us to identify two *RsCLE* genes, *RsCLEm1* and *RsCLEm2*, which are likely to encode proteins with multiple CLE domains and a unique CLE domain composition (Figure 7). There are no identified homologues of these genes in *Arabidopsis*, but they are related to the *B. napus CLEm* genes, which encode multidomain CLE proteins that function as light stimulators of shoot apical meristem activity [15]. The *RsCLEms* contain eight tandem CLE domain sequences and are closely related to *BnCLEm3*, whose product contains five nearly identical tandem CLE domains [15]. 

Thus, in addition to identifying SNVs probably associated with tumours, the sequencing of the radish inbred lines allowed the identification of novel *CLE* family genes.

## 4. Materials and Methods

### 4.1. Plant Material

Closely related lines 18 and 19 of the *R. sativus* genetic collection were used in this study. Both lines originated from a single self-pollinated plant of the Saxa cultivar [9] and are now represented by the progeny of approximately 40–45 generations of inbreeding, indicating an extremely low level of heterozygosity. 

Saxa (cat. № 9464454 in the State Register of Selection Achievements Admitted for Use (National List).) is the cultivar of European radish with round red taproot, which was obtained at the Federal Scientific Centre for Vegetable Growing (Moscow Region, Russia) by the method of mass selection from a sample originating from Central Europe. This radish variety has not yet been subjected to genome sequencing.

### 4.2. Genomic DNA Isolation, Library Preparation, and Sequencing

Total DNA for sequencing was isolated from 50 7-day-old, etiolated radish seedlings of inbred lines according to an unpublished protocol approved by the Laboratory of Plant-Microbial Interactions of the All-Russia Research Institute for Agricultural Microbiology (ARRIAM).

The DNA sequencing of line 19 was performed using Oxford Nanopore technology in the Core Centrum “Genomic Technologies, Proteomics and Cell Biology” at the ARRIAM using a MinION device (Oxford Nanopore, Cambridge, UK). The genome assembly of line 19 was performed using the Canu v.1.7.1 tool (https://github.com/marbl/canu/releases (accessed on 30 April 2024)) with default settings. The sequencing of line 19 was also performed with Illumina technology on the HiSeq2500 sequencer at the Centre of Molecular and Cellular Technologies of Saint Petersburg State University Research Park. The NEBNext^®^ Ultra™ DNA Library Prep Kit for Illumina (New England Biolabs, Ipswich, MA, USA) was used for library construction. Dual barcoding was performed using the NEBNext^®^ Ultra™ DNA Index Prep Kit for Illumina and NEBNext^®^ Multiplex Oligos^®^ Illumina^®^ (Dual Index Primers Set 1). To improve the quality of the genome assemblies, the data were refined to correct possible errors in the Pilon v.1.22 tool (https://github.com/broadinstitute/pilon/releases (accessed on 30 April 2024)) with default settings based on data obtained by two sequencing technologies (Illumina and Nanopore).

The DNA sequencing of line 18 was performed with Illumina technology only, at the Centre of Molecular and Cellular Technologies of Saint Petersburg State University Research Park using the HiSeq2500 sequencer. The NEBNext^®^ Ultra™ DNA Library Prep Kit for Illumina (New England Biolabs) was used for library construction. Dual barcoding was performed using the NEBNext^®^ Ultra™ DNA Index Prep Kit for Illumina and NEBNext^®^ Multiplex Oligos^®^ Illumina^®^ (Dual Index Primers Set 1). Line 18 genome assembly was performed using the SOAPdenovo v.2.04 tool (https://github.com/aquaskyline/SOAPdenovo2 (accessed on 30 April 2024)) with maximal read length = 150, average insert size = 100, cutoff of pair number for a reliable connection = 5).

### 4.3. Bioinformatic Processing of the Sequencing Results

For each assembly, MultiQC v.1.12 [43] and Trimmomatic v.0.40 with the HEADCROP:15 and CROP:140 options [44] were used for quality control and read correction, respectively. The assemblies were indexed using the bowtie2 tool (https://github.com/BenLangmead/bowtie2 (accessed on 30 April 2024)) with default settings. Assemblies of two chromosome-level genomes were generated using the Ragtag tool (https://github.com/malonge/RagTag (accessed on 30 April 2024)) and the chromosome-level reference radish genome GCA_019703475.1 (https://www.ncbi.nlm.nih.gov/data-hub/genome/GCA_019703475.1/ (accessed on 30 April 2024)) with default settings.

Annotation of the genomes of lines 18 and 19 was performed using the Augustus Gene Prediction Tool (https://github.com/Gaius-Augustus/Augustus (accessed on 30 April 2024)) with the *−species = arabidopsis* parameter.

Alignment of the line 19 sequences to the line 18 genome assembly and vice versa was performed using the bowtie2 program (https://github.com/BenLangmead/bowtie2 (accessed on 30 April 2024)), and the identification of candidate genes and differences in the structure of these genes in different radish lines was performed using SnpEff [45], SnpSift [45], and GATK with the HaplotypeCaller option (https://gatk.broadinstitute.org/hc/en-us/articles/360037225632-HaplotypeCaller (accessed on 30 April 2024)), with the parameters SelectVariants --select-type SNP or --select-type INDEL options.

The GO enrichment analysis was performed based on the list of all genes with SNVs and the list of all radish genes as inputs using the R programming language (v. 4.0.2) based on an unpublished custom R script. The GSEABase v. 1.50 (https://bioconductor.riken.jp/packages/3.11/bioc/manuals/GSEABase/man/GSEABase.pdf, accessed on 31 May 2024) was used for data visualisation. A total of 148 pathways related to different biological processes were identified, all of which were statistically significant (p.val_GO ← 0.01, OddsRatio_GO ← 2).

Visualisation of the sequence alignment for assembly and checking for the presence of InDels and SNPs in silico were performed in the IGV genome browser (https://igv.org/ (accessed on 30 April 2024)).

To confirm the detected differences in SNVs between line 18 and line 19 as well as between other tumour (12, 13, 14, 16, 20, 21, 32) and non-tumour (3, 5, 6, 8, 9, 23, 25, 26, 27, 28, 29, 30, 37, 39) lines of the radish genetic collection, DNA was isolated from radish seedlings of the listed lines using the CTAB method. PCR was performed under the following conditions: initial DNA denaturation at 98 °C for 3 min; DNA denaturation at 98 °C for 10 s, primer annealing at 52 °C for 30 s, extension at 72 °C for 1 min, repeated 35 times; and final extension at 72 °C for 5 min. Primers were designed using the VectorNTI software v1.1.1 algorithm (Invitrogen, Waltham, MA, USA) to amplify 300–400-length amplicons and synthesised by Evrogen (Moscow, Russia). The PCR mixtures were subjected to Sanger sequencing.

Sequences for the *RsWOX* genes were searched in the radish genome assemblies represented in the NBCI database (https://www.ncbi.nlm.nih.gov/datasets/genome/?taxon=3725, accessed on 23 January 2024) using the blastP, blastN, and tblastN algorithms of the NCBI database (https://blast.ncbi.nlm.nih.gov/Blast.cgi (accessed on 30 April 2024)), based on the nucleotide and amino acid sequences of *A. thaliana* and *R. sativus* genes and proteins.

The phylogenetic tree of radish CLE protein sequences was constructed based on the alignment of *R. sativus* CLE protein amino acid sequences in MEGA7 software v.10.2. (https://www.megasoftware.net/(accessed on 30 April 2024)) using the Muscle algorithm [46] by Neighbour joining [47] with default parameters and bootstrap 1000 [48]; the tree was visualised using iTOL software v.6.9 (https://itol.embl.de/ (accessed on 30 April 2024)).

Nucleotide and amino acid sequences were analysed using the following programmes: ApE (https://jorgensen.biology.utah.edu/wayned/ape/ (accessed on 30 April 2024), v.3.1.0), SnapGENE (https://www.snapgene.com/ (accessed on 30 April 2024); v.6.0.2), UGENE (http://ugene.net/ru/; v.33), and MEGA7 (https://www.megasoftware.net/ (accessed on 30 April 2024); v. 10.2). Signal motifs were predicted with the SignalP-6.0 tool (https://services.healthtech.dtu.dk/service.php?SignalP (accessed on 30 April 2024)).

The location of genes on radish chromosomes was visualised using the MapChart 2.32 software ((https://www.wur.nl/en/show/mapchart.htm (accessed on 30 April 2024)).

The search for domains in proteins and their visualisation was performed using the MEME online tool (https://meme-suite.org/meme/tools/meme (accessed on 30 April 2024)).

**Figure 8:**
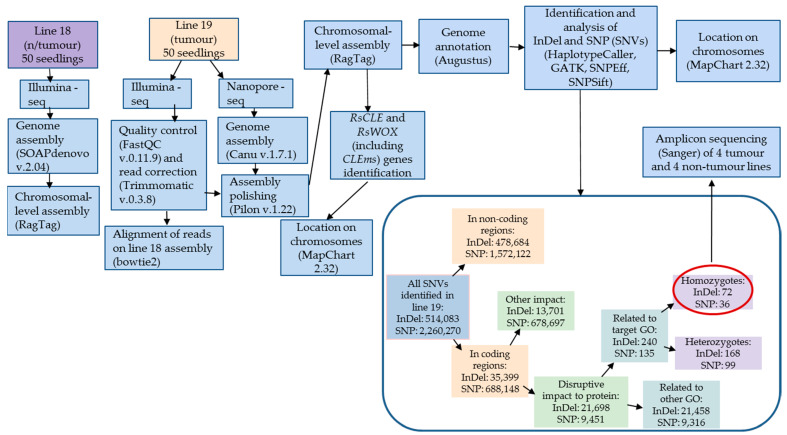
Pipeline of the experiment for the analysis of tumour and non-tumour lines of *Raphanus sativus*. Coloured blocks indicate different stages of this work. SNVs marked with a red circle are the main SNVs investigated in this paper.

All the steps of our experiment are shown graphically in Figure 8.

## 5. Conclusions

By sequencing the genomes of related tumour and non-tumour radish lines, it was possible to identify a number of candidate genes for the role of regulators of spontaneous tumours. Further study of the relationship between the identified genes and tumour formation could increase our knowledge of the role of different pathways involved in the systemic regulation of plant cell division. In addition, this work analysed the *WOX* and *CLE* family genes in radish and identified new, previously unknown *CLE* genes.

## Figures and Tables

**Table 1:** Number of SNVs (InDels, SNPs) identified in the tumour radish line and their probable impacts on gene structure (high, low, moderate, modifier). For SNPs, there is information on their number in different functional classes (missense, nonsense, silent). Data were obtained using the SnpEff tool v.5.1.

Variant		Impact		Functional Class	
Type	Total	Type	Number	Type	Number
SNP	2,260,270	HIGH	9451	MISSENSE	292,963
		LOW	432,159	NONSENSE	5618
		MODERATE	291,254	SILENT	393,274
		MODIFIER	4,334,386		
INDEL	514,083	HIGH	12,234		
		LOW	15,755		
		MODERATE	15,497		
		MODIFIER	1,227,445		

## Data Availability

Data are contained within the article. Raw sequence data are uploaded as BioProject submission SUB13927602.

## Supplementary Materials

The following supporting information can be downloaded at https://www.mdpi.com/article/10.3390/ijms25116236/s1. References [49,50,51,52,53,54,55,56,57,58,59,60,61,62,63,64,65,66,67,68,69,70,71,72,73,74,75,76,77,78,79,80,81,82,83,84,85,86,87,88,89,90,91,92,93,94,95,96,97,98,99,100,101,102,103,104,105,106,107,108,109,110,111,112,113,114,115,116,117,118,119,120,121,122,123,124,125,126,127,128,129,130,131,132,133,134,135,136,137,138,139,140,141,142,143,144,145,146,147,148] are cited in the Supplementary Materials.

## Abbreviations

InDel	Insertion or Deletion
SNP	Single Nucleotide Polymorphism
SNV	Single Nucleotide Variant
