# Supplementary material for: Whole-Genome Sequencing and Analysis of Tumour-Forming Radish (Raphanus sativus L.) Line
Source: Int J Mol Sci. 2024 Jun 5;25(11):6236. doi: 10.3390/ijms25116236 (PMC11172632; doi:10.3390/ijms25116236)
Supplement: Supplementary file 1 [file ijms-25-06236-s001.zip › ijms-3014487-supplementary.pdf]

(a)

Number of effects by type and region

Type

Type (alphabetical order)	Count	Percent
3_prime_UTR_truncation	2	0%
3_prime_UTR_variant	37,044	2.85%
5_prime_UTR_truncation	6	0%
5_prime_UTR_variant	26,901	2.069%
bidirectional_gene_fusion	3	0%
conservative_inframe_deletion	2,784	0.214%
conservative_inframe_insertion	3,798	0.292%
disruptive_inframe_deletion	5,189	0.399%
disruptive_inframe_insertion	4,070	0.313%
downstream_gene_variant	389,820	29.987%
exon_loss_variant	16	0.001%
frameshift_variant	19,300	1.485%
intergenic_region	255,408	19.647%
intron_variant	160,475	12.345%
non_coding_transcript_variant	351	0.027%
splice_acceptor_variant	790	0.061%
splice_donor_variant	1,113	0.086%
splice_region_variant	16,981	1.306%
start_lost	692	0.053%
start_retained_variant	148	0.011%
stop_gained	545	0.042%
stop_lost	239	0.018%
stop_retained_variant	180	0.014%
upstream_gene_variant	374,112	28.779%

Region

Type (alphabetical order)	Count	Percent
DOWNSTREAM	389,809	30.455%
EXON	35,399	2.766%
GENE	3	0%
INTERGENIC	255,408	19.955%
INTRON	143,527	11.214%
SPLICE_SITE_ACCEPTOR	682	0.053%
SPLICE_SITE_DONOR	969	0.076%
SPLICE_SITE_REGION	15,755	1.231%
TRANSCRIPT	351	0.027%
UPSTREAM	374,112	29.229%
UTR_3_PRIME	37,036	2.894%
UTR_5_PRIME	26,880	2.1%

(b)

Number of effects by type and region

Type

Type (alphabetical order)	Count	Percent
3_prime_UTR_variant	103,355	2.024%
5_prime_UTR_premature_start_codon_gain_variant	8,223	0.161%
5_prime_UTR_variant	63,724	1.248%
downstream_gene_variant	1,442,596	28.255%
initiator_codon_variant	96	0.002%
intergenic_region	1,013,932	19.859%
intron_variant	387,403	7.588%
missense_variant	291,254	5.705%
splice_acceptor_variant	1,178	0.023%
splice_donor_variant	1,041	0.02%
splice_region_variant	37,663	0.738%
start_lost	456	0.009%
stop_gained	5,618	0.11%
stop_lost	1,158	0.023%
stop_retained_variant	488	0.01%
synonymous_variant	392,785	7.693%
upstream_gene_variant	1,354,595	26.532%

Region

Type (alphabetical order)	Count	Percent
DOWNSTREAM	1,442,596	28.469%
EXON	688,148	13.58%
INTERGENIC	1,013,932	20.01%
INTRON	356,184	7.029%
SPLICE_SITE_ACCEPTOR	1,178	0.023%
SPLICE_SITE_DONOR	1,041	0.021%
SPLICE_SITE_REGION	34,274	0.676%
UPSTREAM	1,354,595	26.732%
UTR_3_PRIME	103,355	2.04%
UTR_5_PRIME	71,947	1.42%

Figure S1. Effects of different (a). InDels and (b). SNPs on gene structure ranged by type and region. Figure was generated by SnpEff programme.

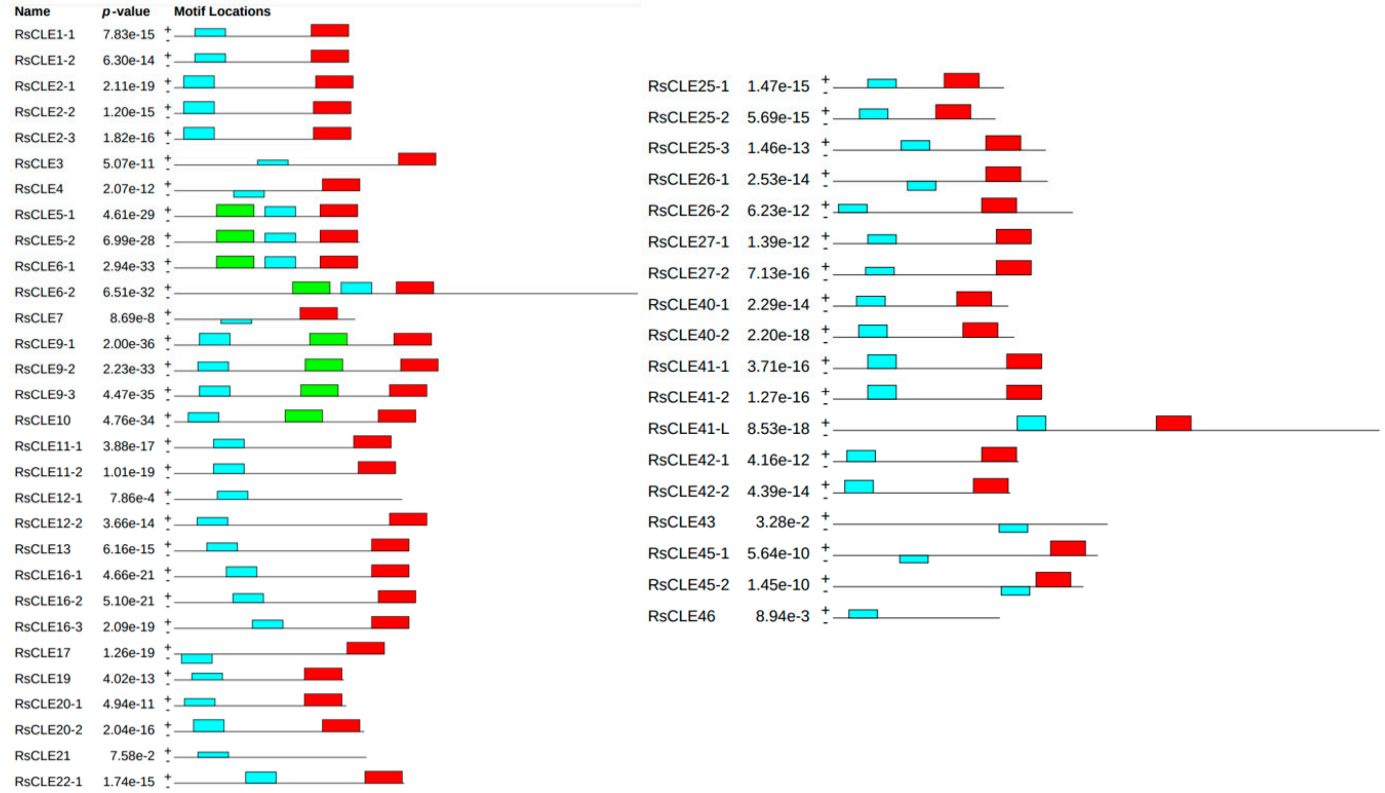

Figure S2. Predicted domains in the structure of the CLE proteins of *R.sativus* line 19. Figure was generated by the MEME online tool (https://meme-suite.org/meme/tools/meme) available on 25.01.2024).

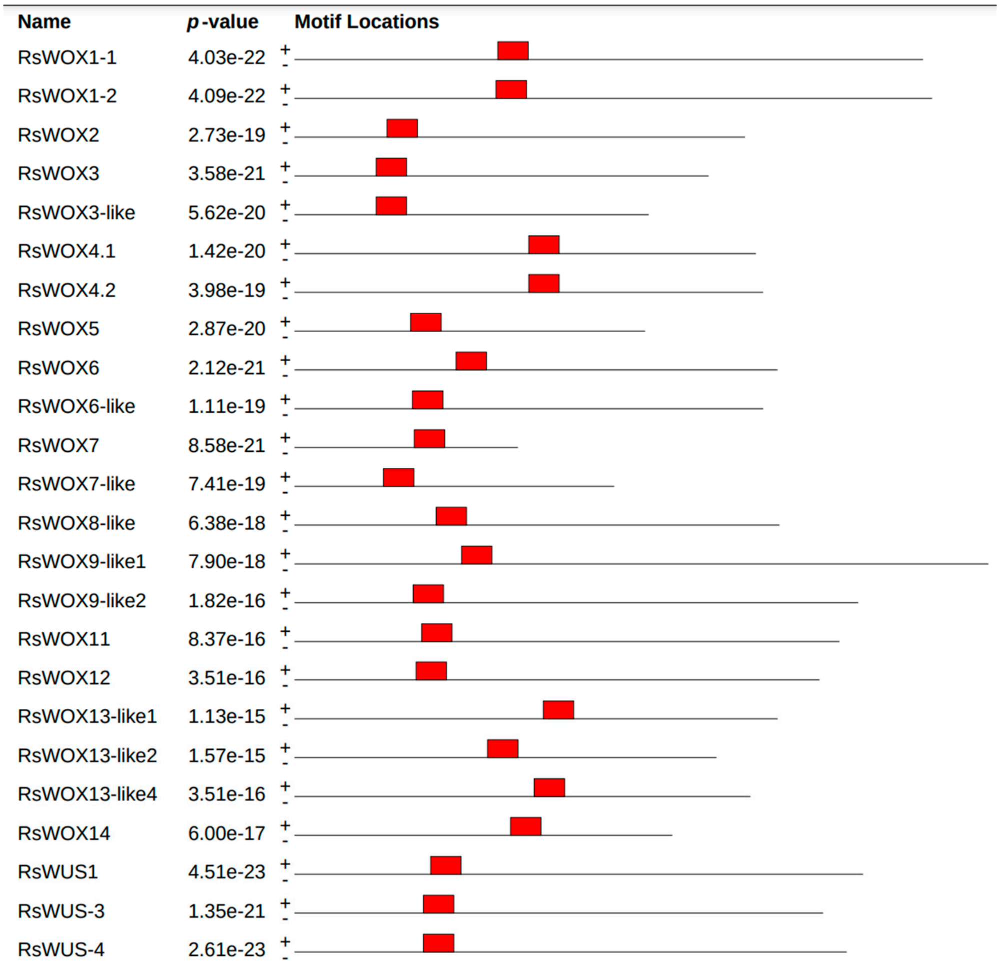

Figure S3. Predicted domains in the structure of the WOX proteins of *R. sativus* line 19. The figure was generated by the MEME online tool ((https://meme-suite.org/meme/tools/meme) available on 25.01.2024).

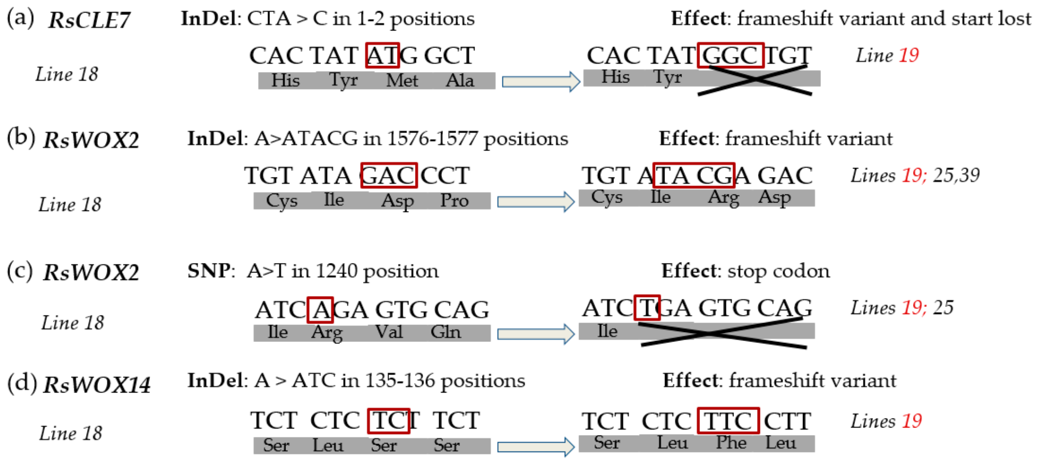

Figure S4. Schematic representation of genes (a). *RsCLE7*, (b), (c). *RsWOX2*, (d). *RsWOX14* without SNVs (left parts of the figure) with SNVs (right parts of the figure) and their likely products. For each SNV, its type and effect on the gene product are indicated. SNVs are highlighted in the red box. Radish tumour lines are highlighted in red.

Table S1. Protein-coding genes with InDels causing disruption of the protein structure in radish tumour line.

№	Gene	Chr	The radish homolog ID in the NCBI database	InDel	Function of *Arabidopsis* homolog	Number of homolo gs in radish	Refere nce
				**Cell cycle control, cell growth and division**			
1	***CFAT***	7	LOC108815961	c.868_871delAGGA|p.Arg290fs	Lignin biosynthesis.	2	[49]
2	***CYCD3;1L***	2	LOC130508373	c.967delC| p.Arg323fs	Cell cycle control during the G1/S transition: activation in response to CK; proliferation of leaf epidermal cells and stomata density; regulation of secondary root growth.	2	[50]
3	***SGO2***	7	LOC108814355	c.391_392insCT|p.Phe131	Cohesion and segregation of sister chromatids in meiosis.	1	[51]
4	***CYCD4;1-L***	9	LOC108828557	c.591_592delCA| p.Lys199fs	Activation of the G1/S transition in response to CK; mitotic cell division; cell cycle activation in RAM; germinal root promotion.	3	[52]
5	***KIN-7N***	6	LOC108807218	c.234_237delGATT|p.Ile79fs	ATP binding; motor function of microtubules.	2	[53]
6	***PDS5***	3	LOC108845796	c.1delA|p.Met1fs	DNA repair; cohesion of sister chromatids in mitosis.	1	[54]
7	***EXPA7L***	6	LOC108810873	c.375_376insTAAT|p.Gly126fs	Weakening of cell walls due to disruption of the bond between cellulose microfibrils and matrix glucans; elongation of root hairs.	3	[55]
8	***EXPA4***	6	LOC108813883	c.1delA|p.Met1fs	Weakening of cell walls due to disruption of the bond between cellulose microfibrils and matrix glucans; formation of syncytium.	3	[56]
9	***LTP1***	4	LOC108853879	c.1dupA|p.Met1fs	Lipid transport across membranes; deposition of wax and cutin in cell walls and secretory tissues; binding of calmodulin.	1	[57]
10	***LOC10881 7684***	7	LOC108817684	c.148dupT|p.Cys50fs	Biosynthesis of xyloglucans.	1	[58]
11	***PME60***	9	LOC108826705	c.2861_2862insCCTC|p.Thr955fs	Modification of cell walls by demethylesterification of pectin.	1	[59]
12	***KIN-4A***	9	LOC108827664	c.61dupA|p.Ile21fs	Deposition of cellulose microfibrils; cell wall mechanics during cell elongation.	2	[60]
13	***LOC10885 9024***	5	LOC108859024	c.2410delG|p.Glu804fs	A gene with unknown function - probably a cell cycle regulator	1	[61]
14	***FZR3***	3	LOC108844659	c.353dupA|p.Gln119fs	Control of ubiquitinligase activity and substrate specificity of anaphase-stimulating complex.	3	[62]
15	***NET1A***	5	LOC108862024	c.1664_1665delTT|p.Val555fs	Association with F-actin at plasma membrane and plasmodesmata; response to pathogen-associated stress; inhibition of root growth.	1	[63]
16	***PCNA1***	5	LOC108832540	c.154_155insGC|p.Leu52fs	Control of DNA replication by increasing polymerase processivity by leading chain elongation.	3	[64]
17	***WOX14***	1	LOC108853278	c.135_136insTC|p.Phe46fs	Functions in the organising centre of the shoot meristem, maintaining stem cells in an undifferentiated state. Together with WOX4 act downstream of PXY, independently regulating plant vascular proliferation.	1	[40]
18	***HAG2***	3	LOC108847241	c.909_910delGG|p.Ala304fs	Histone modification; control of cell cycle, flowering time, response to environmental conditions, hormone signalling and epigenetic processes.	2	[65]
19	***BIN4L***	9	LOC130499962	c.96_100delTTCAT|p.Cys32fs	Chromatin organisation and endoreduplication cycling.	3	[66]

20	***MYB65L***	5	LOC108862100	c.195_196insAGGCTGGGTGCCT|p.Leu66fs	Inhibition of tissue growth by reduced cell proliferation; programmed cell death of protein-storing vacuoles in the aleurone layer during seed germination; anther and tapetum development.	1	[67]
21	***DUF724***	8	LOC108820138	c.25dupA|p.Thr9fs	Polar cell growth.	2	[68]
22	***AGP22***	9	LOC108826274	c.1_1delGA|p.Met1fs	Differentiation, embryogenesis and programmed cell death.	3	[69]
23	***AGP27***	6	LOC108809107	c.111_112delTC|p.Pro38fs	Differentiation, embryogenesis and programmed cell death.	1	[69]
**Control of meristem activity**							
24	***LBD4***	5	LOC108857433	c.17dupG|p.Gly7fs	Phloem/xylem histogenesis; regulation of radial growth through control of cell size and cambium cell divisions.	1	[70]
25	***ASK1***	9	LOC108827710	c.535_536dupTT|p.Leu179fs	Regulation of meristem development; response to osmotic stress.	2	[71]
26	***LOC130500851***	4	LOC130500851	c.654delT|p.Ala219fs	Lateral root initiation and shoot regeneration.	2	[72]
27	***REM15***	6	LOC108808545	c.2917dupT|p.Cys973fs	Flower development.	1	[73]
28	***PLT1***	6	LOC108829093	c.177_178insTA|p.Asp60fs	Specification of the resting root and columella centre; stem cell niche formation during embryogenesis; modulation of polar auxin transport through regulation of PIN gene distribution. A component of the ethylene-activated signalling pathway. Telomere maintenance.	1	[74]
**Regulation of gene expression**							
29	***WOX2***	2	LOC108841333	c.2593delG|p.Glu865fs	Embryonic pattern formation.	1	[75]
30	***ERF121***	8	LOC108837093	c.428_429delAG|p.Lys143fs	Regulation of gene expression by stress factors and components of signal transduction pathways under stress.	6	[76]
31	***ARR21***	7	LOC108815221	c.599delT|p.Leu200fs	B-type response regulator involved in the phosphorelay-type signal transduction system from His to Asp. Activates the A-type RR in response to CK.	2	[77]
32	***SCL23***	4	LOC108848776	c.97delT|p.Ser33fs	Formation of vascular bundle sheath cells.	1	[78]
33	***ERF115-L***	3	LOC108846562	c.232_235delAGCT|p.Ser78fs	Activation of phytosulfokine; rate-limiting of quiescent centre cell division, active when surrounding stem cells are damaged.	3	[79]
34	***bHLH143***	7	LOC108817725	c.182_183dupAA|p.Val62fs	Vascular development.	1	[80]
35	***MYR1***	7	LOC108815809	c.291_294delCCCC|p.Pro98fs	Suppresses flowering and organ elongation when light intensity is reduced; affects gibberellin levels.	1	[81]
36	***PHR1-L1***	9	LOC108824019	c.262_263insTG|p.Ser88fs	Response to phosphorus deficiency; carbohydrate transport.	1	[82]
37	***CDF2***	4	LOC108849692	c.378delA|p.Glu127fs	Control of photoperiodic flowering response through repression of *CONSTANS*.	2	[83]
38	***ARR10***	4	LOC108830348	c.865_866delGC|p.Ala289fs	Activation of RR A-type in response to CK. Callus formation, maintenance of SAM identity, primary root development, regulation of anthocyanin metabolism, chlorophyll biosynthesis, seed growth; response to water deficit.	1	[84]
39	***bZIP19***	8	LOC108818944	c.763_764dupGA|p.Asp255fs	Promotes expression of the zinc transporters ZIP3, ZIP4, ZIP5 and ZIP9 during growth under zinc-deficient conditions.	2	[85]
40	***DIV***	2	LOC108840441	c.38_39dupTG|p.Lys14fs	Suppression of ABA biosynthesis; response to gibberellins; response to salicylic acid; response to salinity.	4	[86]

41	***AGL61***	6	LOC108808389	c.278_279delTG|p.Met93fs	Control of cell differentiation during female gametophyte development.	2	[87]
42	***AGL29***	4	LOC108850931	c.1dupA|p.Met1fs	Pollen development.	2	[88]
43	***bHLH67***	5	LOC108860648	c.632_633insT| p.Thr212fs	Adaptation to cold.	2	[89]
44	***HDG4***	8	LOC108820851	c.1189_1190delGC|p.Ala397fs	Identity of subepidermal cells during postembryonic development.	2	[90]
45	***SCL3***	8	LOC130499224	c.100delA|p.Ile34fs	Response to gibberellins.	1	[91]
46	***AGL24***	4	LOC108852002	c.246_247insC |p.Tyr83fs	Transition to flowering in response to jarrowisation; determination of inflorescence fate in apical meristems; signal transduction via receptor-like kinases. Upon binding to SOC1, mediates the effect of gibberellins on flowering under short-day conditions and regulates LFY expression.	1	[92]
47	***bHLH74-L***	5	LOC108856918	c.1020delT| p.Tyr340fs	Cell elongation; binds to the chromatin region of FT, which promotes its expression and flowering in response to blue light. A component of miR396a -bHLH74 module that regulates root formation in *Arabidopsis* seedlings.	2	[93]
48	***AIL1***	2	LOC130498742	c.1039_1040insTT|p.Tyr347fs	Control of shoot and flower meristems; flowering initiation; seed pod development; flower organ identity; cell proliferation; regulation of gene expression by stress factors.	4	[94]
49	***ERF018***	7	LOC108835321	c.-1_1insCAG	Cell division; response to insect exposure; ethylene-activated signalling pathway; phloem/xylem histogenesis; response to wounding; activation of jasmonate biosynthesis gene expression.	3	[19]
50	***DF1-L***	2	LOC108848832	c.709_710delCC| p.Pro237fs	Suppression of root hair growth; mucilage synthesis in seed coat.	4	[95]
51	***LRL1***	4	LOC108852215	c.206delG|p.Gly69fs	Lipid accumulation under nutrient-deficient conditions; elongation of root hairs.	2	[96]
52	***ERF019***	6	LOC108809887	c.458_459delAA| p.Lys153fs	Response to drought; ethylene-activated signalling pathway; response to fungal infection.	2	[97]
53	***bHLH111***	1	LOC108829884	c.1delA|p.Met1fs	Anthocyanin biosynthesis; expressed in response to nitrogen deficiency	1	[98]
54	***CRF1***	3	LOC108846019	c.252_253insG| p.Thr85fs	Probable target of CLE41; component of CK signalling; seedling, leaf and embryo development; regulation of gene expression by stress factors and components of stress signalling pathways.	1	[99]
55	***CDC5***	8	LOC108830943	c.1494_1495insTT|p.Ala499fs	Regulation of defence responses through control of transcription; mRNA splicing; cell cycle control; SAM development; control of plant innate immunity.	4	[100]
56	***PRHA-L***	8	LOC108821801	c.714dupG|p.Ser239fs	Response to auxin; response to pathogens.	2	[101]
57	***SUVH9-L***	6	LOC108835120	c.865delG|p.Glu289fs	Transposon silencing; RNA-directed DNA methylation.	2	[102]
58	***TOPII***	2	LOC130508706	c.1680delG| p.Arg561fs	Control of DNA topological states by temporary breakage and subsequent reunion of DNA strands.	1	[103]
59	***BBX27***	7	LOC108816763	c.690dupT|p.Ser231fs	Photoperiodic control of short-day flowering.	1	[104]
					**Organogenesis**		
60	***ARA2***	4	LOC108854961	c.926_927insTG| p.Arg310fs	Establishment and development of lateral roots independent of auxin and abscisic acid.	1	[105]
					**Metabolism and signalling of phytohormones**		
61	***CLE7***	4	LOC108853737	c.1_2delAT|p.Met1fs	Signal peptide, synthesised in response to nitrogen deficiency. Expressed in the vascular network and pericycle, inhibits lateral root formation.	1	[106]
62	***SAUR15A-***	3	LOC108846677	c.1dupA|p.Met1fs	Formation of lateral and adventitious roots in response to auxin.	1	[107]

	L						
63	***SAUR32***	6	LOC108809074	c.365_366delAA|p.Lys122fs	Response to auxin; inhibits hypocotyl cell growth.	2	[108]
64	***SAUR40-L***	3	LOC108846468	c.543delA|p.Lys181fs	Response to auxin.	1	[109]
65	***GA2OX3***	4	LOC130510497	c.827_834delCACCATTG|p.Ala276fs	A component of the GC signalling pathway.	1	[110]
66	***LOC10885 7184***	5	LOC108857184	c.72dupT|p.Pro25fs	A gene of unknown function, probably CK-activated	1	
67	***HK5-L***	3	LOC108844370	c.2573delA|p.Asn858fs	Negative regulation of ETR1-dependent ABA and ethylene signalling pathway; inhibits root elongation; opening/closing of stomata in closing cells by ABA-independent pathway; control of AFC production in response to stress.	3	[111]
68	***ETR1***	7	LOC108817715	c.77dupT|p.Leu26fs	A two-component negative regulator of ethylene signalling. Control of seed germination, control of RAM.	1	[112]
**Other functions**							
69	***LOC10884 2019***	2	LOC108842019	c.716_717insAT|p.Leu240fs	A putative receptor kinase.	2	[113]
70	***PNG1***	6	LOC108813526	c.1773_1779delGCCGCCA|p.Pro592f s	Protein quality control.	2	[114]
71	***LOC10885 1456***	4	LOC108851456	c.383_384delTT|p.Phe128fs	Binding of metal ions; DNA replication.	1	[115]
72	***ASP39***	6	LOC108812392	c.387_388insC| p.Lys130fs	Synthesis of phytochelatins and homophytochelatins that bind heavy metals. Degradation of glutathione conjugates.	1	[116]

Table S2. Protein-coding genes with SNPs causing disruption of the protein structure in radish tumour line.

№	Gene	Chr	The radish homolog ID in the NCBI database	SNP	Function in radish	Number of homologs in radish	Reference
**Cell cycle control**							
1	***CYCJ18***	4	LOC108830438	c.718T>C| p.Ter240Argext*	Cell division; regulation of cyclin-dependent protein serine/threonine kinase activity; phase transition of the mitotic cell cycle.	1	[117]
2	***PS1L***	9	LOC108861742	c.1297G>T| p.Glu433*	Orientation of the division spindle in the second division of meiosis, formation of microspore tetrads.	1	[118]
3	***AUR1***	2	LOC108819297	c.1168C>T| p.Arg390*	Cytokinesis; formation of microtubule organisation centres.	2	[119]
4	***bZIP1***	5	LOC108825955	c.64C>T|p.Arg22*	Spatial control of cytokinesis by proper assembly of the phragmoplast.	1	[120]
5	***LRX1***	5	LOC108836486	c.1638C>A| p.Tyr546*	Cell morphogenesis, including root hairs, through cell wall formation and assembly and/or growth polarisation.	3	[121]
6	***AGP18***	4	LOC108807296	c.578C>A| p.Ser193*	Differentiation, embryogenesis, and programmed cell death.	3	[122]
7	***COBL11***	2	LOC108841217	c.574A>T| p.Arg192*	Pollen tube formation and development.	1	[123]
**Metabolism and signalling of phytohormones**							
8	***GRR1-L1***	9	LOC108825013	c.665A>T| p.Ter222Leuext*	Reception of auxin; response to auxin.	3	[124]
**Regulation of gene expression**							
9	***CHR35***	5	LOC108862654	c.2327T>A| p.Leu776*	Epigenetic control of RNA-directed DNA methylation.	1	[125]
10	***CMT1***	1	LOC108807343	c.902G>C| p.Ter301Ser*	CpXpG methylation; gene silencing.	1	[126]
11	***WOX2***	2	LOC108841333	c.1204A>T| p.Arg402*	Embryonic pattern formation.	1	[75]
12	***bZIP44***	7	LOC108814136	c.415T>C| p.Ter139Glnext*	Regulation of seed germination by loosening of micropylar endosperm and seed coat rupture.	2	[127]
13	***ARF21***	9	LOC108822118	c.487A>T| p.Lys163*	Response to auxin.	1	[128]
14	***NAC017***	5	LOC108856330	c.2T>C|p.Met1	Response to stress.	1	[129]
15	***MYB104L***	6	LOC108810575	c.448C>T| p.Gln150*	Microgametogenesis, development of polarised microspores.	1	[130]
16	***NF-YB8***	3	LOC108844481	c.566A>T| p.Ter189Leuext*	Control of flowering time; flavonoid biosynthesis.	1	[131]
17	***WRKY55L***	3	LOC130509746	c.363G>A| p.Trp121*	Regulation of leaf senescence through control of salicylic acid and ROS accumulation.	2	[132]
18	***ERF119***	4	LOC108833429	c.841T>G| p.Ter281Gluext*	Regulation of gene expression by stress factors and components of stress-mediated signal transduction pathways; formation of secondary cell wall.	3	[133]

19	***ARR21***	3	LOC108846809	c.1694C>G | p.Ser565*	B-type response regulator involved in a phosphorelay-type signal transduction system from His to Asp. Activates A-type RR in response to CK.	2	[77]
20	***REM5***	2	LOC130494531	c.392G>T | p.Ter131Leut*	Vesicular transport.	2	[134]
21	***bHLH63***	2	LOC108824102	c.487A>T | p.Lys163*	Epigenetic regulation of flowering: activation of FT expression, flowering in response to blue light. Suppression of the innate immune response.	2	[135]
22	***AGL80***	2	LOC108815229	c.432A>G | p.Ter144Trp*	Endosperm cell proliferation.	1	[136]
23	***SHOC1***	5	LOC130500734	c.20T>A | p.Leu7*	Biosynthesis of gibberellins; seed germination; differentiation of xylem elements, lignification of secondary cell wall.	1	[137]
24	***CDF3***	6	LOC108813543	c.984G>A | p.Trp328*	Photoperiodic control of flowering through suppression of CONSTANS; enhances nitrogen use efficiency; increases biomass production and yield under salinity stress.	1	[138]
25	***dZIP30***	4	LOC108848413	c.240T>A | p.Cys80*	TF, a repressor of reproductive development, meristem size and plant growth. Interacts with regulators of meristem and gynoecium development.	1	[139]
				**Control of meristem activity**			
26	***REM10L***	1	LOC108857973	c.604A>T | p.Lys202*	Flower development.	2	[73]
27	***BRX***	5	LOC108850063	c.946T>A | p.Ter316Arg*	Proliferation and elongation of root and shoot cells; control of the protofloem; interactions between ISC and brassinosteroid signalling; CK-mediated inhibition of lateral root initiation.	1	[140]
				**Other functions**			
28	***PER30***	5	LOC108862038	c.2T>C | p.Met1	Oxidation of toxic reducing agents; lignin biosynthesis and degradation, auxin catabolism, response to exogenous stresses.	1	[141]
29	***CRWN***	1	LOC108806851	c.8G>A | p.Trp3*	Organisation of core structure.	1	[142]
30	***LRR-RK***	2	LOC108842145	c.1526T>A | p.Leu509*	Encodes protein with serine/threonine/tyrosine kinase activity.	1	[143]
31	***LecRLK***	9	LOC108824077	c.107G>Tp.Ter36Leuext	Plant development; response to stress.	1	[144]
32	***SBT2.6***	4	LOC108849971	c.2442C>G | p.Tyr814*	Proteolysis; activated in response to eoCLE40.	1	[145]
33	***LOC108853 023***	4	LOC108853023	c.87T>A | p.Tyr29*	Participant in ubiquitin-dependent catabolic processes.	2	[146]
34	***VLG***	6	LOC130496156	c.309C>G | p.Tyr103*	Gametogenesis	1	[147]
35	***LOC108856 505***	5	LOC108856505	c.1354C>T | p.Arg452*	Response to hypoxia.	1	[148]
36	***LOC108854 537***	1	LOC108854537	c.3G>A | p.Met1	No data.	1	
